# Latent-profile analysis of sleep disturbances, cognitive performance and neuropsychiatric symptoms reveals subtypes of Parkinson’s disease

**DOI:** 10.3389/fneur.2026.1765246

**Published:** 2026-03-04

**Authors:** Lyna Mariam El Haffaf, Magdalena Eriksson Domellöf, Lucas Ronat, Oury Monchi, Lois Walton, David Bäckström, Carl-Johan Boraxbekk, Lars Forsgren, Lars Nyberg, Anna Stigsdotter Neely, Jarkko Johansson

**Affiliations:** 1 Centre de Recherche de l’Institut Universitaire de Gériatrie de Montréal, Montreal, QC, Canada; 2 Faculty of Art and Science, Department of Psychology, University of Montreal, Montreal, QC, Canada; 3 Department of Psychology, Umeå University, Umeå, Sweden; 4 Faculty of Medicine, Department of Neurosciences, University of Montreal, Montreal, QC, Canada; 5 Faculty of Medicine, Department of Radiology, Radio-Oncology, and Nuclear Medicine, University of Montreal, Montreal, QC, Canada; 6 Department of Social and Psychological Studies, Karlstad University, Sweden; 7 Department of Clinical Science, Neurosciences, Umeå University, Umeå, Sweden; 8 Faculty of Medical and Health Sciences, Institute for Clinical Medicine, University of Copenhagen, Copenhagen, Denmark; 9 Department of Neurology, Institute of Sports Medicine Copenhagen (ISMC), Copenhagen University Hospital Bispebjerg, Copenhagen, Denmark; 10 Umeå Functional Brain Imaging (UFBI) Lab, Umeå University, Umeå, Sweden; 11 Department of Health, Education, and Technology, Luleå University of Technology, Luleå, Sweden; 12 Department of Diagnostics and Intervention, Umeå University, Umeå, Sweden

**Keywords:** [^11^C]-raclopride PET, cognitive performance, neuropsychiatric symptoms, Parkinson’s disease, sleep disturbances

## Abstract

**Objective:**

Given the clinical heterogeneity of Parkinson’s disease (PD), identification of early -stage subgroups with shared non-motor symptom (NMS) profiles may clarify its pathophysiology. This study used latent-profile analyses (LPA) to define subgroups based on sleep disturbances, cognitive performance and neuropsychiatric symptoms, and examined dopaminergic function and brain volume differences between them.

**Methods:**

We analyzed data from 51 cognitively normal non-PD older adults and 105 early-stage PD participants from the iPARK trial, including 19 who underwent [^11^C]-raclopride PET/MR. Participants completed the Hospital Anxiety and Depression Scale, the short version of the Karolinska Sleep Questionnaire and a battery of neuropsychological tests. LPA were used in PD to identify subgroups based on NMS profiles, which were then characterized and examined in relation to dopaminergic integrity and brain morphology.

**Results:**

LPA identified a two-cluster solution as the best fit. Group 1 (*N* = 49) showed poorer working memory, executive function and processing speed along with greater daytime sleepiness, depression and anxiety. Group 2 (*N* = 56) exhibited less affected cognitive function and minimal NMS. Groups were similar in demographics, disease duration, motor symptom severity and medication, but differed on UPDRS-1 NMS. Group 1 demonstrated significantly reduced [^11^C]-raclopride binding potential compared to Group 2 in the left putamen at both ROI- and voxel-wise analysis.

**Conclusion:**

These findings indicate clinically distinct subgroups in early-stage PD. Greater NMS burden is linked to impaired dopaminergic integrity, suggesting a potential neurobiological signature. Early identification of such subgroups may improve understanding of disease heterogeneity and support personalized management and interventions.

**Clinical trial registration:**

https://clinicaltrials.gov/study/NCT03680170?id=NCT03680170&rank=1, identifier (NCT03680170).

## Introduction

Parkinson’s disease (PD) has traditionally been conceptualized as a movement disorder with diagnosis relying on the presence of motor symptoms (1). However, a growing body of research has highlighted the complex and heterogeneous nature of PD, involving a broad range of non-motor symptoms (NMS). Among these, sleep disturbances, cognitive impairment and neuropsychiatric symptoms, such as depression and anxiety, are highly prevalent, frequently co-occurring and often precede the onset of motor symptoms (2–4). They significantly impact quality of life, increase caregiver burden and are major contributors to hospitalization and early institutionalization, often more so than motor symptoms (5, 6).

Sleep disturbances affect approximately 60% to 98% of patients with PD from disease onset (7) and encompass a wide range of issues including insomnia, excessive daytime sleepiness (EDS) and rapid eye movement sleep behavior disorder (RBD) (8, 9). In PD, the presence of sleep disturbances is associated with greater level of depression and anxiety, both cross-sectionally and longitudinally (10–13). For instance, Zhu et al. (14) identified depression and anxiety as key determinants of sleep quality. In longitudinal studies, Rutten et al. (15) reported a bidirectional relationship between insomnia and anxiety, while Amara et al. (13) found that EDS was associated with both depression and anxiety. Sleep disturbances have also been associated with cognitive impairment in individuals with PD. Studies have shown that they increase the risk of developing dementia in PD (16, 17), and are closely associated with impairments in global cognition, working memory, executive functions and attention (18). Similarly, depressive and anxious symptoms have been linked to deficits in executive functioning and memory (5, 19). The overlapping associations between these NMS of PD are further supported by van Rooden et al. (20) who identified four clinical clusters in PD using exploratory factor analysis. One of these was characterized by the co-occurrence of EDS, depression, cognitive impairment, autonomic dysfunction, psychosis and axial symptoms, while another included sleep disturbances, depression and cognitive impairment. These findings support the view that sleep disturbances in PD are not isolated but tend to appear in specific combinations and may be linked to distinct clinical profiles.

Thus, identifying distinct patient subgroups based on their NMS profiles could offer valuable insights into early disease heterogeneity and its underlying pathophysiological mechanisms. However, the neural basis of these symptom associations remains poorly understood (5). One proposed explanation is that disruption of fronto-striatal circuit may underlie sleep disturbances, cognitive impairments and neuropsychiatric symptoms, providing a common neuroanatomical substrate between them (21–24). Nonetheless, neuroimaging studies have produced inconsistent and sometimes contradictory findings, making it difficult to identify clear structural or functional brain correlates (25).

Given the complexity and heterogeneity of NMS presentation in PD, data-driven approaches, such as latent-profile analysis (LPA), offer a powerful method to identify meaningful subgroups and explore their underlying neural mechanisms. These approaches allow phenotypic profiles to emerge directly from the data, free of *a priori* assumptions, by grouping patients to maximize between-group differences while minimizing within-group variability (26). Although previous studies have classified PD patients based on various NMS combinations (27–29), these approaches differ in the specific symptoms assessed and the methods used, limiting comparability across studies. Importantly, few studies have examined the associated pathophysiological mechanisms, which represents a key contribution of the present work. In this study, we applied LPA to identify subgroups of individuals with early-stage PD based on sleep disturbances, cognitive performance and neuropsychiatric symptoms. We then characterized these subgroups and examined whether they differed in dopaminergic integrity and brain structures.

## Materials and methods

### Participants

The iPARK trial is a double-blinded, randomized controlled trial with a parallel-group design. The overall objective is to investigate the effect of a process-based cognitive training program that focuses on working memory updating, compared to a low dose short-term memory paradigm in participants with PD (30).

We extracted the data of 156 participants at baseline, specifically: 105 PD participants with PD and 51 cognitively normal non-PD older adults (CN). Informed consent was obtained from each participant at pre-test. Inclusion criteria for participants with PD were: (1) age between 45 and 75 years; (2) a confirmed diagnosis of PD according to the United Kingdom Parkinson’s Disease Brain Bank; (3) a pathological Dopamine Transport single-photon emission computed tomography (DAT-SPECT) scan; (4) stable dopaminergic medication for the past 3 months; (5) a Mini-Mental State Examination (MMSE) of 24 or higher; (6) being between stages one and three on the Hoehn and Yahr stage; and (7) no dementia diagnosis. Diagnosis of PD and Hoehn and Yahr assessments were established by neurologist specialized in movement disorders. The exclusion criteria for the study are as follows: (1) unstable medication, (2) ongoing cognitive training, (3) diagnosis of dementia, (4) drug or alcohol abuse and (5) other disease of the central nervous system or other serious medical condition.

The CN participants were recruited between October 2022 and November 2024 through local ads seeking healthy individuals aged 55–75 for a study on cognition and aging. A telephone screening excluded individuals with self-reported medical or psychological conditions affecting cognition (e.g., neurological and psychiatric disorders) as well as those with other circumstances that could hinder participation in the cognitive testing (e.g., impaired hearing or vision).

The dataset generated and analyzed for the current study is not publicly available due to the ethical Review Act but is available from the corresponding author on reasonable request. The iPARK trial has been approved by the Swedish Ethical Review Authority in Sweden (DNR: 2018-351-32M).

### Clinical assessment

Baseline clinical information [disease severity, most affected side, global cognitive function (MMSE), DAT-SPECT, Hoehn and Yahr stage, non-motor aspects of experiences of daily living (Unified Parkinson’s Disease Rating Scale (UPDRS) part I; (31))] and motor function (UPDRS part III) were collected from the participant’s medical journals. All other data were collected on paper forms and/or computer programs.

A battery of neuropsychological test was administered to assess participants’ cognitive abilities. These include the Trail Making test A and B (TMT-A and TMT-B; DKEFS), the perdue pegboard, the digit symbol, the digit span (WAIS IV), the spatial span, the letter memory running span, the digit memory running span, the Stroop test (DKEFS) and the Buschke SRP (30).

To assess sleep, the short form of the Karolinska Sleep Questionnaire (KSQ-short) was given to all participants (32). The KSQ-short is a self-assessment form that measures different aspects of sleeping difficulties over the past month. Participants are asked to answer six questions on a 5-point Likert scale from 1 = never/very good to 5 = always/very bad. The Parkinson’s disease questionnaire (PDQ-39) assesses health-related quality of life, including functioning and well-being, in individuals with PD. For the present study, only question 30 of the PDQ-39 was used to assess the presence of EDS. Participants had to answer the question “Due to having Parkinson’s disease, how often during the last month have you unexpectedly fallen asleep during the day?” on a Likert scale from 0 = never to 4 = always.

Neuropsychiatric symptoms of depression and anxiety were assessed using the Hospital Anxiety Depression Scale (HADS). This is a 14-item long questionnaire equally targeting anxiety and depression. Responses are rated on a 0–3 Likert scale (0 = never/no problems and 3 = always/a lot of problems) and analyses were based on the separate depression and anxiety subscale scores.

Other demographic variables include sex, years of education, age at baseline, age of PD onset and the levodopa equivalent daily dose (LEDD).

### Brain imaging: acquisition and analysis

PET/MR measurements were recorded from 19 PD participants at baseline with a 3T General Electric Signa PET-MR system with a 15-channel head coil.

#### Regional volumes

Structural T1-weighted images were obtained with the following acquisition parameters: FOV: 25 × 20 cm^2^, Matrix: 256 × 256, Slice Thickness: 1 mm, Slices: 180, TE: 3.1 ms, TR: 7,200 ms, Flip Angle:12, Bandwidth: 244.1 Hz/Pixel. Freesurfer version 7.4.1 was used to process T1-weighted images and derive estimates of gray matter volume. Subcortical gray matter segmentations and cortical parcellations based on the Desikan-Killiany atlas were used to define regions of interest (ROIs) (33). We extracted total cortical and subcortical gray matter volume as well as bilateral prefrontal, temporal, caudate and putamen volumes (34, 35). The prefrontal volumes included the bilateral superior, rostral middle and caudal middle frontal regions, the lateral and medial orbitofrontal regions, the pars *opercularis, triangularis* and *orbitalis*, the rostral and caudal anterior cingulate regions and the frontal pole. The temporal volumes included the bilateral entorhinal, fusiform, inferior, middle and superior temporal regions as well as the parahippocampal region, the temporal pole and the transverse temporal region. For each ROI, we used a linear regression model to assess group differences while controlling for estimated total intracranial volume (eTIV). Multiple comparisons were corrected across all models using the Benjamini-Hochberg false discovery rate (FDR) procedure.

#### [^11^C]-raclopride PET scan

All participants were injected with a bolus (250 MBq) of [^11^C]-Raclopride following the local standard protocols for [^11^C]-Raclopride PET studies (36) to measure binding potential (BP) for striatal D2 receptors. PET imaging was conducted over 55 min from tracer injection. Participants executed a working memory task between 40 and 55 min, while the first 40 min after injection was measured at resting-state. Therefore, a reconfigured multi-linear reference tissue model (MRTM) was used to estimate distinct binding potentials during resting-state and task execution using cerebellar cortex as reference tissue (37, 38). Here, we only used the resting-state binding potential to assess the D2 receptor availability at rest.

Preprocessing of PET data was conducted in Statistical Parametric Mapping (SPM, version 12, Wellcome Institute, London, UK) software. First, frame-to-frame co-registration was conducted to compensate for head motion during PET scanning. Second, a sum image of motion corrected PET data was registered to T1-MRI, and all PET data was re-sliced to match T1-orientation and voxel size. Regional time-activity course (TAC) data were extracted using the mean voxel-wise radioactivity data within each ROI. Finally, regional and voxel-wise TAC data were submitted to modified MRTM to estimate resting-state binding potential non-displaceable (BP_ND_).

### Statistical analysis

Normality was assessed using skewness and kurtosis statistics and the Shapiro–Wilk test. Variables showing non-normal distributions underwent Winsorizing to limit the influence of outliers (39). For variables that remained non-normal after Winsorizing, non-parametric statistical tests were applied. Only variables with 5% missing values or less were retained for the PCA and clustering analyses. Missing data within these variables were handled using group-wise mean imputation to preserve sample size and reduce bias (39).

Statistical analyses were performed on the open-source web application Jupyter Notebook (version 6.4.11) using scripts written in Python language (3.9.12). To reduce dimensionality and derive data-driven cognitive domains, we conducted a principal component analysis (PCA) with varimax rotation on standardized (z-scored) neuropsychological test scores, computed relative to the PD sample mean and standard deviation. This approach extracts latent constructs that capture shared variance across measures, enhancing interpretability and minimizing redundancy (40, 41). Components were retained based on loadings, explained variance and conceptual clarity. Varimax rotation was applied to facilitate clearer factor interpretation and rotated components scores were computed by projecting standardized test scores onto the rotated loading matrix. To enable direct comparison between groups, the same PCA solution and PD-derived scaler were applied to the CN group, allowing their cognitive score to be projected onto the same component space.

In addition, we performed a PCA with varimax rotation on standardized (z-scored) KSQ-short items. This approach was informed by Nordin et al. (32), who identified two key dimensions of sleep quality and non-restorative sleep, based on the same set of items used in our analysis. Replicating this structure allowed us to capture core sleep domains while minimizing variable redundancy. PCA was first applied to the PD group to derive a population-specific sleep profile, then the solution was projected onto the CN group for comparison. Component loadings and explained variance were reported to support interpretation.

We conducted a Latent-Profile Analysis (LPA) using the *GaussianMixture* function from the scikit-learn library (version 1.0.2) in Python on PD participant only, to identify and characterize heterogeneity within PD. This method models the data as a mixture of Gaussian distributions, estimating the probability that each observation belongs to a latent class (26). LPA was conducted using Gaussian Mixture Modeling (GMM) framework. Although classical LPA typically assumes conditional independence among indicators, GMM offers a more flexible estimation approach. In this manuscript, the term LPA is used to describe the conceptual latent-variable approach aimed at identifying distinct profiles, rather than to denote a specific estimation algorithm. A series of models with 1 to 4 classes were conducted to identify latent subgroups representing distinct multivariate patterns of sleep, cognitive and neuropsychiatric variables. Each class captures the joint distribution of the variables, representing a profile of participants with similar patterns. Model fit was evaluated using the Akaike Information Criterion (AIC), with lower AIC values indicating better model fit. AIC was selected because it has been shown to perform well in smaller samples [<500; (42)]. However, we acknowledge that alternative indices may favor different solutions, which represent a limitation when interpreting the results. We also report the Bayesian Information Criterion (BIC), entropy and average posterior probabilities for each solution. The optimal number of profiles was chosen based on the lowest AIC and BIC, entropy values and average posterior probabilities exceeding 0.80, combined with consideration of theoretical interpretability and sufficient class size (26).

Variables included in the analysis were PCA scores for cognitive and sleep domains, the standardized z-scores from PDQ-39 item 30 (assessing excessive daytime sleepiness) and the HADS depression and anxiety subscales. Interpretation of the results was based on the average scores of the measures. We examined differences among the PD groups in terms of age, disease duration, most affected side, years of education, MMSE scores, UPDRS-I, UPDRS-III, LEDD and sex distribution. To do so, we performed a Mann–Whitney U test for continuous variable and a Chi-Square test for categorical variable of sex and most affected side. We also used a Mann–Whitney U test to compare each LPA groups to the CN group, in order to assess the extent to which each PD group differs from healthy participants. We further examined the differences among the PET/MR subgroups using Mann–Whitney U test for continuous variable and a Ficher’s exact test for categorical variable. Descriptive statistics are reported as median and interquartile range (IQR; 25th–75th percentile). The rank-biserial correlation (r_rb_) was calculated as a measure of effect size, with values <0.3 interpreted as small, 0.3–0.5 as medium, and >0.5 as large effects. To assess the robustness of findings given the modest PET subsample size, bootstrap resampling was performed. Finally, the PET/MR subsample was also compared to the full LPA-derived groups to confirm that it retained similar demographic and clinical profiles, supporting its representativeness for subsequent neuroimaging analyses. All analyses were subjected to a False Discovery Rate (FDR) correction. Corrections were applied using the Python library statsmodels (version 0.13.2), specifically the function stats.multitest.fdrcorrection with the Benjamini/Hochberg method and a family-wise error rate (FWE) threshold of 0.05.

## Results

### Principal component analysis

We retained four components for the cognitive PCA, which collectively explained 75.1% of total variance. Component loadings revealed four distinct cognitive domains: Component 1 (PC1) correlated with working memory (Spatial Span, Digit Updating, Letter Memory test); Component 2 (PC2) captured episodic memory (Buschke immediate and delayed recall); Component 3 (PC3) represented processing speed (TMT-A, Perdue Pegboard and Digit Symbol); Component 4 (PC4) reflected executive functioning (Stroop and TMT shifting cost). PC varimax rotation and the explained variance for each component are detailed in Table 1.

**Table 1:** Factor loading after varimax rotation for cognitive tests.

Cognitive test	PC1 – Working memory	PC2 – Memory	PC3 – Processing speed	PC4 – Executive function
Spatial span	**−0.397**	−0.080	0.115	0.016
Digit updating test	**−0.567**	0.028	0.023	0.047
Letter memory test	**−0.578**	0.030	−0.143	−0.145
Buschke recall	−0.074	**−0.690**	−0.010	0.023
Buschke delayed	0.044	**−0.713**	0.014	−0.007
Digit symbol - WAIS	−0.084	−0.028	**0.447**	−0.194
TMT-A	0.310	−0.039	**−0.465**	−0.146
Perdue Pegboard	0.237	0.002	**0.731**	0.015
Stroop	−0.100	0.043	0.061	**0.778**
TMT – shift cost	0.098	−0.053	−0.112	**0.559**

Explained variance: PC1: 0.447; PC2: 0.130; PC3: 0.093; PC4: 0.081.

Bold indicates variables loading on the same principal component.

In accordance to Nordin et al. (32), we accepted two component for the KSQ-short, which together explained 71% of the total variance. Component 1 (PC1) reflected sleep quality and included the following items: frequent awakening with difficulty falling back asleep, waking up too early and disturbed/restless sleep. Component 2 (PC2) captured non-restorative sleep, characterized by difficulty falling asleep, difficulty waking up and not feeling rested upon waking. Rotated loading and explained variance for each component are shown in Table 2.

**Table 2:** Factor loading after varimax rotation for the KSQ.

KSQ	PC1 – Sleep quality	PC2 – non-restorative sleep
a. Difficulty falling asleep	0.149	**0.467**
b. Difficulty waking up	−0.397	**0.728**
c. Frequent awakenings with difficulty falling back asleep	**0.518**	0.105
d. Not feeling rested upon waking	0.247	**0.430**
e. Waking up too early	**0.574**	−0.058
f. Disturbed/restless sleep	**0.402**	0.229

Explained variance: PC1- sleep quality: 0.495; PC2-non-restorative sleep: 0.210.

Bold indicates variables loading on the same principal component.

### Latent-profile analysis

**Figure 1:**
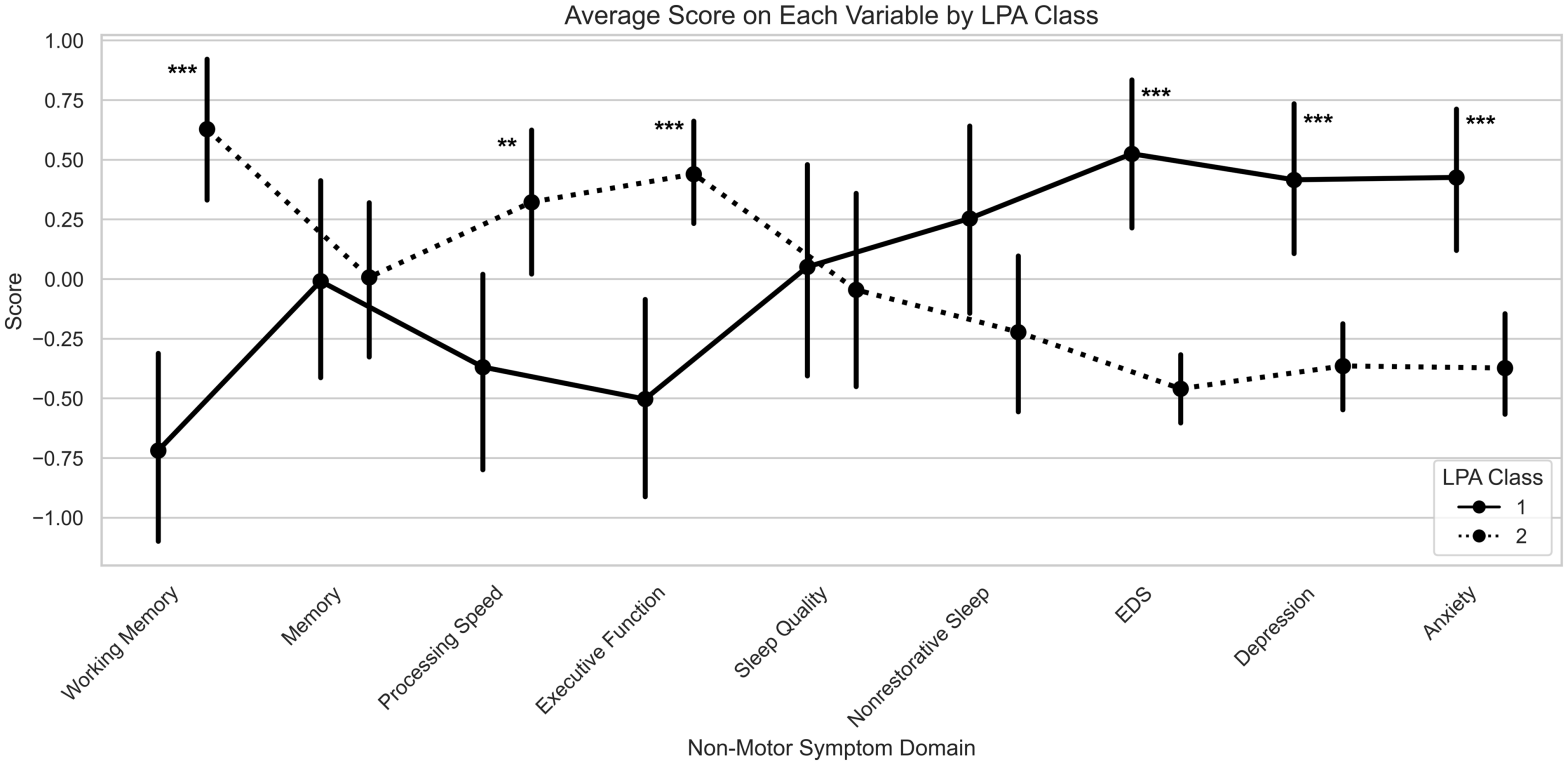
Mean profiles of non-motor symptoms of Parkinson’s disease for the 2-class solution. EDS, excessive daytime sleepiness question from the PDQ-39. Significant differences between groups are indicated with asterisks: *p < 0.05; **p < 0.01; ***p < 0.001.

Latent-profile models specifying 1, 2, 3 or 4 classes for cognitive and sleep domains, PDQ-39 item 30 and HADS depression and anxiety were estimated. The model fit indices are reported in Table 3. Model fit indices converged on the 2-class solution. AIC was minimized at 2 classes (AIC = 2939.1), while BIC was lowest for 2-classes (BIC = 3228.4), compared to the 3-class (BIC = 3421.1) and 4-class solutions (BIC = 3590.9). The 2-class solution demonstrated excellent classification quality, with entropy of 0.93 and average posterior probability of 0.98, both exceeding the recommended threshold of 0.80 (26). Although alternative 3-class and 4-class solutions produced higher entropy values, they were not selected due to substantially higher AIC and BIC values, indicating worse overall model fit. Thus, our results show a clear two class solution within the PD cohort, which was considered the best fit to the data. We referred to them as Group 1 and Group 2. Figure 1 shows the profiles of mean cognitive, sleep and neuropsychiatric symptoms scores for the 2-class solution. Group 1 (*n* = 49; 46.7%) was characterized by poorer performance in working memory, processing speed and executive function along with greater daytime sleepiness, depression and anxiety. In contrast, Group 2 (*n* = 56; 53.3%) exhibited less affected cognitive function and minimal NMS symptoms.

**Table 3:** Model fit indices.

Fit indices	One class	Two classes	Three classes	Four classes
Log-Likelihood	−1435.7	−1360.5	−1328.9	−1285.8
AIC	2979.4	2939.1	2985.9	3009.7
BIC	3122.7	3228.4	3421.1	3590.9
Entropy	–	0.93	0.94	0.96
AvgPP	–	0.98	0.96	0.97

AIC, akaike information criterion; BIC, Bayesian information criterion; AvgPP, average posterior probability.

### Demographic characteristics

Descriptive analyses showed no significant differences in terms of age, sex, disease duration, most affected side, motor symptom severity (UPDRS-III) or LEDD between Group 1 and Group 2. However, we observed a significant difference in UPDRS-I scores (U = 446.0, *p* = 0.00018, p_fdr_ = 0.0016). This aligns with our interpretation that Group 1 exhibits more pronounced NMS than Group 2 (Table 4).

**Table 4:** Demographic characteristics at baseline based on the 2-class solution from the latent profile analysis.

Variables	Group 1 (*N* = 49)	Group 2 (*N* = 56)	Chi-square/U	*p*	p_fdr_
Female, *n* (%)	21 (42.9%)	26 (46.4%)	0.03	0.86	0.91
Male, *n* (%)	28 (57.1%)	30 (53.6%)			
Age (years)	65.6 ± 7.0	64.8 ± 6.9	1244.0	0.41	0.62
Disease duration (years)	4.4 ± 2.8	3.5 ± 2.2	1089.0	0.087	0.26
Education (years)	13.4 ± 2.9	14.2 ± 2.7	1560.5	0.22	0.40
MMSE score	29.2 ± 1.2	29.0 ± 1.1	892.0	0.65	0.83
UPDRS1 score	2.8 ± 1.8	1.4 ± 1.5	446.0	**0.0002**	**0.002**
UPDRS3 score	14.6 ± 7.7	12.7 ± 6.9	1165.5	0.19	0.40
LEDD	563.5 ± 367.0	495.2 ± 260.1	1353.0	0.91	0.91
Most affected side					
Left, *n* (%)	24 (49%)	17 (30.3%)	5.0	0.083	0.26
Right, *n* (%)	22 (46%)	30 (53.6%)			
Both, *n* (%)	3 (6%)	9 (16.1%)			

Mann–Whitney U test for continuous variables and Chi-square test for categorical variable of sex and most affected side. Group 1 = PD-NMS+; Group 2 = PD-NMS−; MMSE, Mini-Mental State Evaluation; UPDRS1, Unified Parkinson’s Disease Rating Scale I; UPDRS3, Unified Parkinson’s Disease Rating Scale III; LEDD, Levodopa equivalent dosage; *p*, *p*-values. p_fdr_, FDR corrected p-values. Bold font indicates *p*-values <0.05.

To determine whether group differences were solely driven by UPDRS-I scores, we performed regression analysis predicting LPA variables, with group membership as the main predictor while controlling for age, disease duration, sex, years of education, LEDD, UPDRS-I and UPDRS-III. The group effect remained significant for working memory, executive function and EDS (assessed via PDQ-39 item 30), whereas memory, processing speed, sleep quality and non-restorative sleep showed no significant group difference, consistent with expectations. Notably, depression ( β = − 0.38, *p* = 0.073) and anxiety ( β = − 0.42, *p* = 0.055) scores lost significance after controlling for UPDRS-I, indicating that these symptoms are largely accounted for within the broader NMS severity measured by UPDRS-I. This additional finding further suggests that the LPA-derived groups capture meaningful cognitive and sleep-related differences beyond the general non-motor symptom burden.

We further characterized the subgroup of PD participants that underwent PET/MR scanning. No significant difference among the two PET/MR subgroups was found in terms of sex, age, disease duration, education, MMSE score, UPDRS-I, UPDRS-III and LEDD. A significant group difference was found for the most affected side (U = 16.67, *p* = 0.041), but it did not remain significant after FDR correction (p_fdr_ = 0.37; Table 5).

**Table 5:** Demographic characteristics at baseline based on the 2-class solution from the latent-profile analysis for the PET/MR subsample.

Variables	Group 1 (*N* = 8)	Group 2 (*N* = 11)	F/U	*p*	p_fdr_
Female, *n* (%)	4 (50.0%)	5 (45.5%)	0.8	0.99	0.99
Male, *n* (%)	4 (50.0%)	6 (54.5%)			
Age (years)	65.6 ± 6.7	64.2 ± 5.9	20.0	0.55	0.99
Disease duration (years)	3.3 ± 2.2	3.8 ± 3.5	17.5	0.84	0.99
Education (years)	13.5 ± 1.7	12.5 ± 2.5	20.5	0.42	0.99
MMSE score	29.0 ± 0.9	29.0 ± 0.9	13.5	0.72	0.99
UPDRS1 score	1.2 ± 0.8	1.3 ± 0.7	16.5	0.99	0.99
UPDRS3 score	9.3 ± 3.5	10.4 ± 3.9	13.5	0.73	0.99
Most affected side					
Left, *n* (%)	5 (62.5%)	1 (9.1%)	16.7	**0.041**	0.37
Right, *n* (%)	3 (37.5%)	10 (90.9%)			
Both, *n* (%)	0 (0%)	0 (0%)			
LEDD	414.9 ± 109.1	367.6 ± 183.1	15.0	0.93	0.99

Mann–Whitney U test for continuous variables and Fisher’s exact test for categorical variable of sex and most affected side. Group 1, PD-NMS+; Group 2, PD-NMS−; MMSE, Mini-Mental State Evaluation; UPDRS1, Unified Parkinson’s Disease Rating Scale I; UPDRS3, Unified Parkinson’s Disease Rating Scale III; LEDD, Levodopa equivalent dosage; *p*, *p*-values; p_fdr_, FDR corrected *p*-values.

Bold font indicates p-values < 0.05.

To confirm the validity of the LPA-derived classification within the imaging subsample, we compared this subsample to the full LPA-derived groups. This comparison ensures that the subgroups maintain their distinct demographic and clinical profiles in the imaging cohort, supporting its representativeness for subsequent neuroimaging analyses. No significant differences were found between the imaging subsample of Group 1 and the full LPA-derived Group 1. This suggests that the subsample is representative of the larger group in terms of demographic and clinical characteristics. When comparing the imaging subsample to the remainder of Group 2, a significant difference in years of education was observed (U = 53.5, *p* = 0.007), although this difference did not remain significant after FDR correction (p_fdr_ = 0.081).

### Comparison between CN and LPA groups

Descriptive analysis revealed no significant difference between CN and Group 1 as well as between CN and Group 2 in terms of sex, years of education, MMSE scores, memory domain performance, sleep quality or non-restorative sleep (Table 6). Group 1 showed significantly worse working memory (U = 1828.0, *p* < 0.001, p_fdr_ = 0.001), processing speed (U = 1932.0, *p* < 0.001, p_fdr_ < 0.001) as well as higher depression (U = 467.0, *p* < 0.001, p_fdr_ < 0.001) and anxiety scores (U = 688.0, *p* < 0.001, p_fdr_ = 0.001) compared to CN. In contrast, Group 2 differed from CN only in processing speed (U = 1923.0, *p* = 0.002, p_fdr_ = 0.007) and depression scores (U = 922.5, *p* = 0.001, p_fdr_ = 0.006). Overall, this indicates that Group 1 was particularly characterized by working memory and anxiety differences relative to the other groups. Finally, the CN group was significantly older than Group 2 (U = 1937.5, *p* = 0.001, p_fdr_ = 0.007).

**Table 6:** Characteristics at baseline based on the 2-class solution from the latent profile analysis and the CN group.

Variables	Group 1 (*N* = 49)	Group 2 (*N* = 56)	CN (*N* = 51)	Chi-square/U (G1 vs CN)	p (G1 vs CN)	p_fdr_ (G1 vs CN)	Chi-square/U (G2 vs CN)	p (G2 vs CN)	p_fdr_ (G2 vs CN)
Female, *n* (%)	21(42.9%)	26 (46.4%)	29 (56.9%)	1.4	0.23	0.36	0.8	0.38	0.53
Male, *n* (%)	28(57.1%)	30 (53.6%)	22 (43.1%)						
Age (years)	65.6 ± 7.0	64.8 ± 6.9	69.0 ± 3.7	1556.5	**0.034**	0.099	1937.5	**0.001**	**0.006**
Education	13.4 ± 2.9	14.2 ± 2.7	14.5 ± 2.7	1473.0	0.12	0.22	1475.5	0.77	0.92
MMSE	29.1 ± 1.1	29.0 ± 1.1	29.3 ± 0.9	1122.0	0.52	0.66	1324.5	0.24	0.36
Working memory	−0.7 ± 1.4	0.6 ± 1.2	0.5 ± 1.3	1828.0	**<0.001**	**0.001**	1315.0	0.48	0.64
Memory	−0.009 ± 1.5	0.008 ± 1.3	0.5 ± 1.3	1537.0	**0.048**	0.12	1715.0	0.074	0.16
Processing speed	−0.4 ± 1.5	0.3 ± 1.2	1.0 ± 1.1	1932.0	**<0.001**	**<0.001**	1923.0	**0.002**	**0.007**
Executive function	−0.5 ± 1.5	0.4 ± 0.8	0.05 ± 0.9	1430.0	0.22	0.36	1093.0	**0.037**	0.099
Sleep quality	0.05 ± 1.6	−0.05 ± 1.6	0.03 ± 1.3	1234.5	0.92	0.95	1458.0	0.85	0.94
Nonrestorative sleep	0.3 ± 1.5	−0.2 ± 1.3	−0.2 ± 1.2	1000.5	0.087	0.17	1457.0	0.86	0.94
Depression score	0.4 ± 1.2	−0.4 ± 0.7	−0.8 ± 0.6	467.0	**<0.001**	**<0.001**	922.5	**0.001**	**0.006**
Anxiety score	0.4 ± 1.0	−0.4 ± 0.8	−0.3 ± 0.9	688.0	**<0.001**	**0.001**	1417.5	0.95	0.95

Mann–Whitney U test for continuous variables Chi-square test for categorical variable of sex. Group 1 (G1), PD-NMS+; Group 2 (G2), PD-NMS−; MMSE, Mini-Mental State Evaluation; *p*, *p*-values; p_fdr_, FDR corrected *p*-values. Bold font indicates *p*-values *p* < 0.05.

### Differences in binding potential and brain volumes among groups

**Figure 2:**
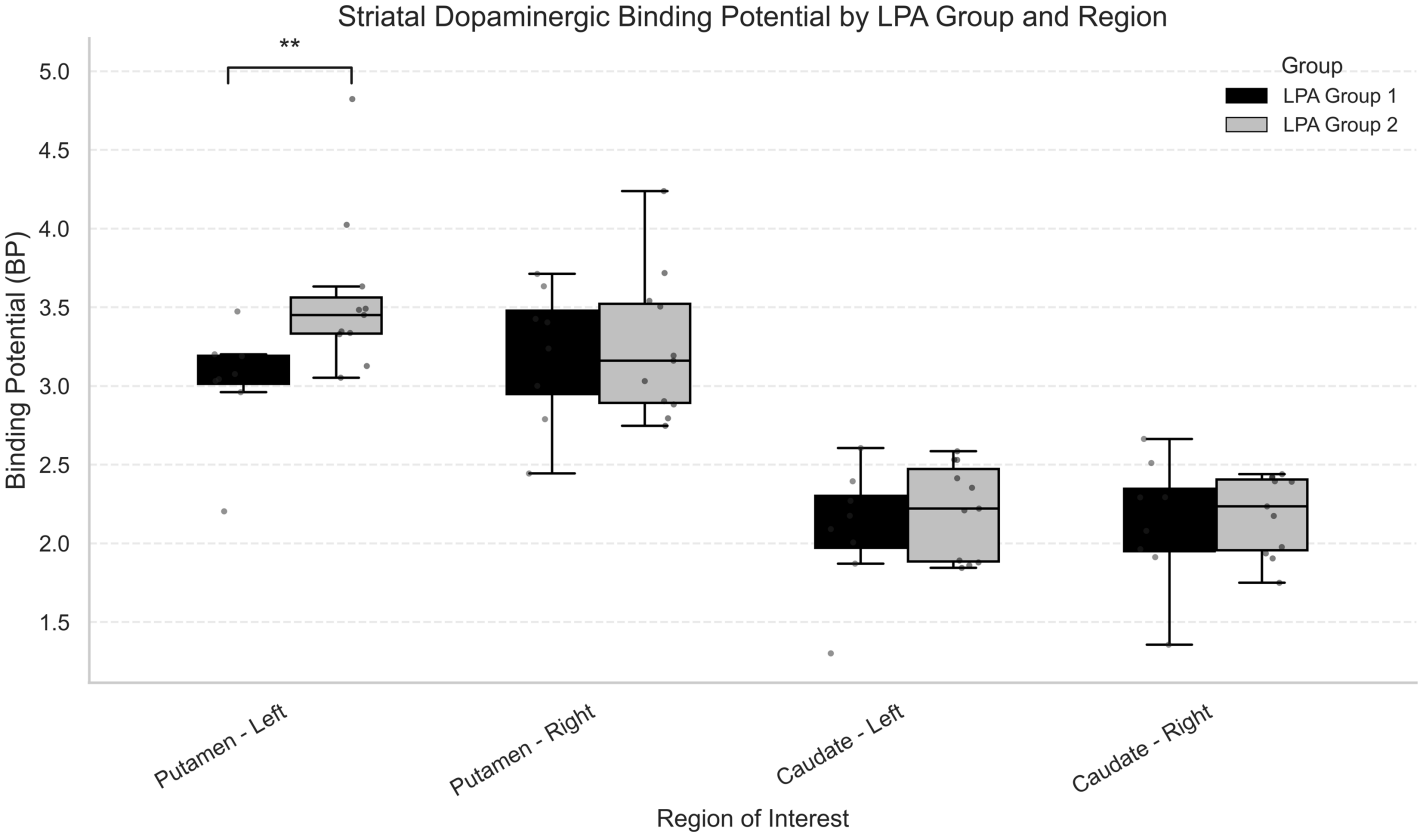
Regional binding potential across LPA groups. ** indicates a significant difference between groups in the left putamen.

**Figure 3:**
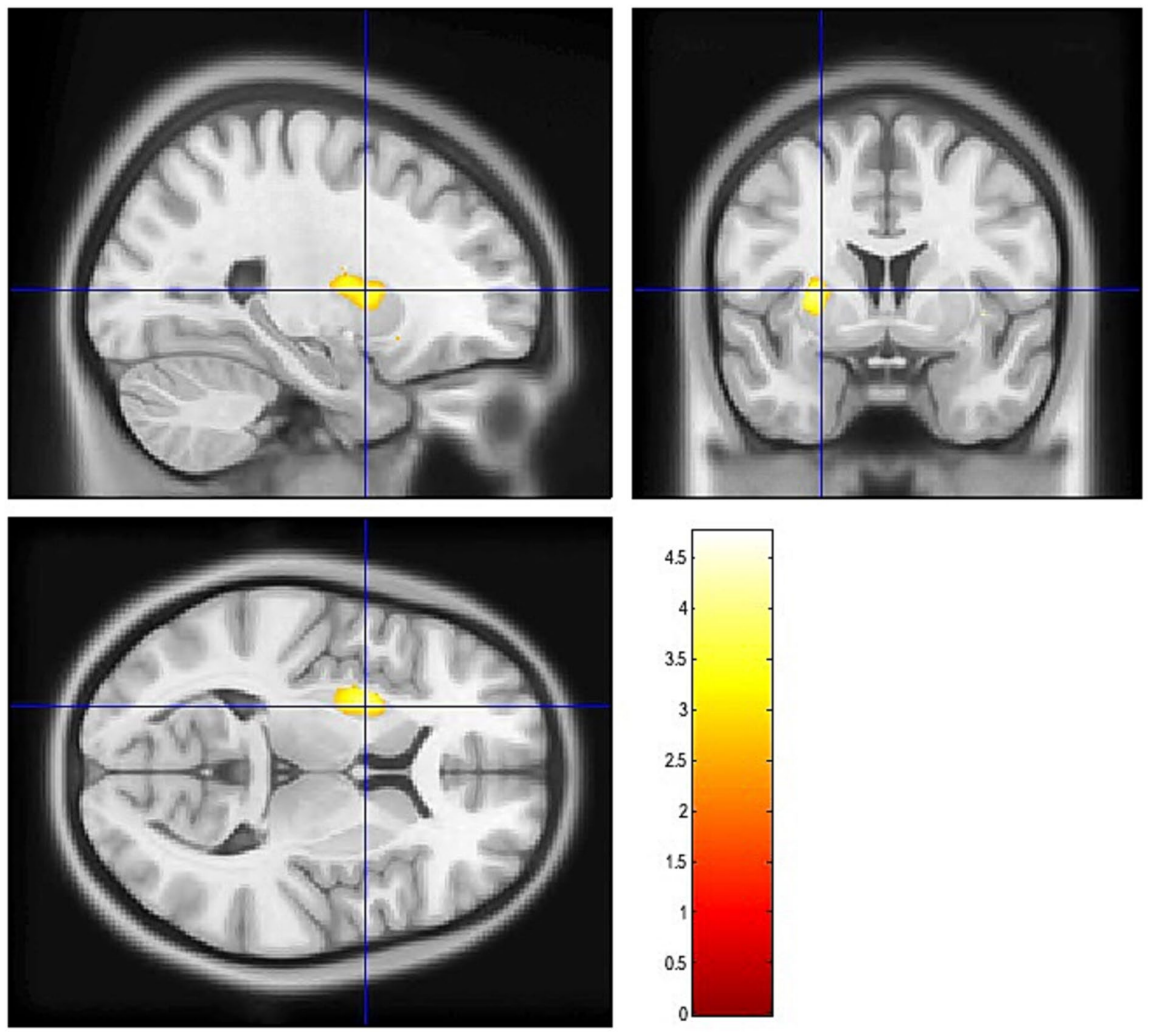
Significant PET-derived Voxel clusters in the left putamen.

Group 1 demonstrated significantly reduced [^11^C]-raclopride binding potential in the left putamen at both ROI (U = 11.0, *p* = 0.005, p_fdr_ = 0.02; Table 7; Figure 2) and voxel-wise analyses (Figure 3), with a large and robust effect size [r_rb_ = 0.75, bootstrap 95% CI (0.39: 1.00)]. This suggests lower D2 receptor availability in this region for Group 1 compared to Group 2. No significant differences were observed in total cortical and subcortical volumes as well as in the caudate and the putamen (Table 8). Significant differences in cortical volume were observed in the left rostral anterior cingulate ( β = − 391.6, *p* = 0.024, p_fdr_ = 0.30), frontal pole ( β = − 171.2, *p* = 0.047, p_fdr_ = 0.30) and fusiform gyrus ( β = − 829.3, *p* = 0.027, p_fdr_ = 0.34), with group 2 showing lower volumes compared to group 1. However, these results did not remain statistically significant after FDR correction.

**Table 7:** Group differences in binding potential at baseline.

Regions of interest	U	Group 1 Median (IQR)	Group 2 Median (IQR)	Median diff	95% CI Lower	95% CI Upper	r_rb_	Bootstrap 95% CI Lower	Bootstrap 95% CI Upper	*p*	p_fdr_
Putamen – Right	44.0	3.32 (0.53)	3.16 (0.63)	0.16	−0.50	0.54	0.00	−0.57	0.50	0.99	0.99
Putamen – Left	11.0	3.06 (0.18)	3.45 (0.23)	−0.39	−0.60	−0.14	0.75	0.39	1.00	**0.005**	**0.02**
Caudate – Right	43.0	2.19 (0.40)	2.23 (0.45)	−0.05	−0.45	0.36	0.02	−0.52	0.61	0.97	0.99
Caudate – Left	38.0	2.13 (0.33)	2.22 (0.59)	−0.09	−0.48	0.38	0.14	−0.43	0.68	0.66	0.99

Mann–Whitney U test for continuous variables. Group 1, PD-NMS+; Group 2, PD-NMS−. Data presented as median (IQR), where IQR = 25th–75th percentile range. Mean diff = Group 1 median minus Group 2 median, negative values indicate lower binding in Group 1; 95% CI = 95% confidence interval for median difference; r_rb_ = rank-biserial correlation (<0.3 small, 0.3–0.5 medium, >0.5 large); Bootstrap 95% CI = 95% confidence interval for effect size from 10,000 bootstrap resamples; *p*, *p*-values; p_fdr_, FDR corrected *p*-values. Bold font indicates *p*-values *p* < 0.05.

**Table 8:** Group differences in brain volume controlling for estimated total intracranial volume.

Region	β	SD	*t*	*p*	p_fdr_	95% CI	r ^2^
Total Gray	−9293.3	10302.1	−0.9	0.38	0.87	[−31132.7 to 12546.1]	0.79
Subcortical Gray	−549.5	1648.9	−0.3	0.74	0.89	[−4045.0 to 2946.07]	0.50
Right Caudate	−301.8	183.7	−1.6	0.12	0.80	[−691.2 to 87.6]	0.19
Left Caudate	−241.0	182.2	−1.3	0.20	0.80	[−627.3 to 145.3]	0.11
Right Putamen	−195.1	202.2	−0.96	0.35	0.87	[−623.6 to 233.5]	0.34
Left Putamen	−277.7	232.6	−1.2	0.25	0.80	[−770.7 to 215.3]	0.27
Right Caudal Anterior Cingulate	264.5	157.4	1.7	0.11	0.73	[−69.1 to 598.1]	0.27
Left Caudal Anterior Cingulate	218.4	203.2	1.1	0.30	0.90	[−212.5 to 649.3]	0.07
Right Caudal Middle Frontal	179.5	406.5	0.4	0.66	0.91	[−682.3 to 1041]	0.06
Left Lateral Middle Frontal	−272.4	292.5	−0.9	0.37	0.90	[−892.6 to 347.8]	0.20
Right Lateral Orbito- Frontal	−55.4	268.4	−0.2	0.84	0.91	[−624.5 to 513.6]	0.53
Left Lateral Orbito- Frontal	130.8	252.6	0.5	0.62	0.90	[−404.6 to 666.2]	0.40
Right Medial Orbito- Frontal	29.7	244.4	0.1	0.9	0.91	[−488.5 to 547.8]	0.47
Left Medial Orbito- Frontal	−374.1	278.8	−1.3	0.19	0.86	[−965.0 to 216.9]	0.40
Right Paracentral	−35.0	162.0	−0.2	0.83	0.91	[−378.4 to 308.3]	0.15
Left Paracentral	158.9	263.4	0.6	0.55	0.90	[−399.5 to 717.5]	0.26
Right Pars Opercularis	53.2	458.4	0.1	0.91	0.91	[−918.5 to 1024.9]	0.13
Left Pars Opercularis	−112.9	434.3	−0.3	0.80	0.90	[−1033.6 to 807.8]	0.05
Right Pars Orbitalis	128.9	146.1	0.9	0.39	0.91	[−180.7 to 438.5]	0.21
Left Pars Orbitalis	11.6	93.1	0.1	0.9	0.90	[−185.6 to 208.9]	0.09
Right Pars Triangularis	119.4	327.2	0.4	0.72	0.91	[−574.2 to 813.0]	0.13
Left Pars Triangularis	−95.2	277.4	−0.3	0.74	0.90	[−683.2 to 492.8]	0.02
Right Precentral	−129.5	788.2	−0.2	0.87	0.91	[−1800.3 to 1541.4]	0.12
Left Precentral	−312.7	616.36	−0.5	0.62	0.90	[−1619.3 to 994.0]	0.32
Right Rostral Anterior Cingulate	−98.9	201.2	−0.5	0.63	0.91	[−525.5 to 327.7]	0.098
Left Rostral Anterior Cingulate	−391.6	157.4	−2.5	**0.024**	0.30	[−725.3 to −57.9]	0.5
Right Rostral Middle Frontal	−823.1	809.6	−1.0	0.32	0.91	[−2539.3 to 893.1]	0.47
Left Rostral Middle Frontal	166.8	912.5	0.2	0.86	0.90	[−1767.6 to 2101.3]	0.25
Right Superior frontal	−1426.1	770.7	−1.9	0.083	0.73	[−3060.0 to 207.9]	0.42
Left Superior Frontal	312.8	984.4	0.3	0.75	0.90	[−1765.0 to 2408.5]	0.11
Right Frontal Pole	−27.3	95.0	−0.3	0.78	0.91	[−228.6 to 174.1]	0.28
Left Frontal Pole	−171.2	79.5	−2.2	**0.047**	0.30	[−339.7 to −2.8]	0.34
Right Entorhinal	−111.5	226.7	−0.5	0.63	0.94	[−1220.2 to 3626.7]	0.03
Left Entorhinal	58.7	210.3	0.3	0.78	0.94	[−389.6 to 506.9]	0.04
Right Fusiform	−617.2	509.4	−1.2	0.24	0.94	[−1703.0 to 468.6]	0.55
Left Fusiform	−829.3	338.6	−2.4	**0.027**	0.34	[−1551.1—107.5]	0.47
Right Inferior Temporal	248.3	584.6	0.4	0.68	0.94	[−997.7 to 1494.3]	0.39
Left Inferior Temporal	−279.9	459.6	−0.6	0.55	0.94	[−1259.5 to 699.6]	0.69
Right Middle Temporal	−75.3	571.4	−0.1	0.90	0.94	[−1293.3 to 1142.6]	0.38
Left Middle Temporal	−139.6	622.3	−0.2	0.82	0.94	[−1466.1 to 1186.8]	0.40
Right Parahippocampal	−28.2	75.8	−0.4	0.72	0.94	[−189.6 to 133.3]	0.03
Left Parahippocampal	52.3	107.6	0.5	0.63	0.94	[−177.0 to 281.5]	0.21
Right Superior Temporal	91.5	499.1	0.2	0.86	0.94	[−972.4 to 1155.4]	0.27
Left Superior Temporal	243.6	621.9	0.4	0.70	0.94	[−1082.0 to 1569.1]	0.26
Right Temporal Pole	161.5	144.0	1.1	0.23	0.94	[−145.4 to 468.5]	0.10
Left Temporal Pole	229.4	206.7	1.1	0.28	0.94	[−211.1 to 669.9]	0.17
Right Transverse Temporal	56.0	58.0	1.0	0.35	0.94	[−67.7 to 179.7]	0.40
Left Transverse Temporal	20.9	103.4	0.2	0.84	0.94	[−199.5 to 241.4]	0.04

Linear model estimates of group effect on brain volume. β , coefficient; SD, standard error; *t*, *t*-value; *p*, *p*-value; p_fdr_, FDR corrected *p*-values; 95% CI, 95% confidence interval. Bold font indicates *p*-values *p* < 0.05.

## Discussion

In this study, we identified and characterized subgroups of early PD participants using data-driven methods applied to cognitive, affective and behavioral measures. We then examined the underlying pathophysiology of these profiles with regards to dopaminergic integrity and brain volumes. Building on previous work that have identified heterogenous clinical subtypes in early PD, our study is among the first to integrate these domains within a single data-driven framework to explore associated pathological mechanisms.

We identified two-cluster solution as the best fit, with Group 1 showing poorer performance in working memory, processing speed and executive function along with greater daytime sleepiness, depression and anxiety. Group 2, in contrast, exhibited less affected cognitive function and minimal NMS. These results align with prior studies. Notably, Erro et al. (27) described a non-motor dominant cluster exhibiting high NMS score, intermediate levels of depression, anxiety and frontal cognitive impairment as well as intermediate UPDRS-III scores, features consistent with our affected Group 1. Similarly, Mavandadi et al. (43) reported a subgroup with poorer cognitive performances accompanied by more severe psychiatric symptoms, while other studies, focusing primarily on cognitive measures, identified executive and frontal dysfunction subtypes (28, 29, 44). Importantly, our two groups did not differ in age, sex, education, disease duration, global cognition (MMSE), motor severity or LEDD, indicating that they do not simply represent different disease stages or confounding demographic effects. Thus, our findings extend this literature by demonstrating that, when sleep, cognitive and neuropsychiatric symptoms are jointly considered, they converge to define a distinct multi-symptom phenotype in early PD.

Group 2, defined by minimal cognitive and non-motor disturbances, is highly consistent with the intact or slight NMS presentation described in previous studies (27–29, 43). Thus, this group can be considered a distinct clinical subtype, characterized by a lower burden of non-motor symptoms, which likely translates to a better quality of life and a slower rate of disease progression in these domains (45). Together, these patterns converge to reinforce the crucial concept of phenotypic heterogeneity in PD, demonstrating that patients do not follow a uniform disease trajectory. The present study focused on cognitive, affective and behavioral measures, extending this notion to a previously uninvestigated constellation of NMS indicators.

To explore whether the clustering of these NMS arises from shared neural mechanism, we examined dopaminergic integrity. Group 1 demonstrated significantly reduced [^11^C]-raclopride binding potential in the left putamen at both ROI- and voxel-wise analyses. Although putamen dysfunction is typically associated with motor impairment in PD, converging evidence highlight its role in NMS such as sleep disturbances (46–48), neuropsychiatric symptoms (49–51), and cognitive impairments (52–54). For instance, in early, drug-naïve PD participants, sleep disturbances have been linked to lower dopamine transporter (DAT) uptake in the bilateral striatum and both the anterior and posterior putamen (46). Similarly, two [^123^I] FP-CIT SPECT studies reported correlations between subjective EDS and dopamine transporter loss in the striatum, caudate and putamen (47, 48). Affective symptoms have also been tied to left-lateralized dopaminergic dysfunction. Weintraub et al. (49) reported reduced DAT availability in the left anterior putamen in PD patients with depression and anxiety, especially early in the disease course. Similarly, regarding cognitive impairments, Fiorenzato et al. (52) found that attention and executive performance (symbol digit modality test) correlated with DAT binding in the left putamen and bilateral caudate. They also noted that left-hemisphere dopaminergic denervation was linked to greater cognitive impairment at onset and during progression, whereas right hemisphere deficits related more closely to motor severity. Beyond global executive deficits, the left striatum has also been implicated more specifically in working memory updating. Dahlin et al. (55) demonstrated that activation of the left striatum mediated transfer effects of updating training, underscoring its involvement in this core working memory process. In line with this, Backman et al. (56) reported that working memory training induced increased striatal dopamine release, highlighting the dopaminergic sensitivity of these associative circuits. Thus, the reduced dopaminergic integrity in the left putamen observed in Group 1 may be associated with not only to broader executive deficits, but also selective vulnerabilities in working memory updating. This interpretation is in line with the functional organization of the striatum, where the putamen participates in both motor loops and associative and limbic circuits that support cognitive and emotional regulation (57). Accordingly, one of the primary objectives of the iPARK project was to shed light on the dopaminergic mechanisms of working memory in PD pre- and post- working memory training (30).

Taken together, these findings are consistent with our observation of reduced [^11^C]-raclopride BP in the left putamen in Group 1, suggesting a potential association between dopaminergic integrity in this region and a specific constellation of NMS in early PD. This neurobiological distinction is particularly notable as the groups were matched on key clinical variables, including LEDD, motor symptoms severity (UPDRS-III) and disease duration. These group differences align with previous research showing that early cognitive impairment, a feature of Group 1, defines a PD subgroup with a distinct and more severe prognosis. Consistent with this, Backstrom et al. (30) found that cognitive impairment, rather than motor severity, is among the strongest early predictor of mortality in PD. In this context, our findings suggest that left putamen dopaminergic dysfunction might be linked to a worse trajectory with more unfavorable cognitive and survival outcomes, refining our understanding of disease heterogeneity. Finally, our results align with emerging evidence of hemispheric specialization, whereby left-sided dopaminergic dysfunction is more strongly implicated in sleep, cognitive and affective disturbances (46, 49, 52).

Given this pattern of left-sided dopaminergic dysfunction, with its hypothesized links to associative and limbic cortico-striatal networks, might be expected to coincide with broader cortical and prefrontal volumetric changes. However, we found no significant differences in total cortical or subcortical volumes and in prefrontal and temporal volumes, after correction for multiple comparisons. Structural MRI studies in PD without dementia have yielded inconsistent results, whereas volumetric changes are more reliably observed in PD with dementia (58). Nevertheless, we observed trend-level reductions in the cortical volumes of the left rostral anterior cingulate, frontal pole and fusiform gyrus. These results may hint at early structural alterations within associative and limbic cortices. The absence of robust volumetric differences, combined with a left-sided dopaminergic alteration in Group 1, is consistent with the hypothesis that early circuit-level dopaminergic disruptions may precede measurable cortical atrophy, though this temporal sequence requires confirmation through longitudinal investigation. Such a dissociation is consistent with the hypothesis that subcortical dopaminergic differences may be associated with non-motor symptomatology in PD independently of measurable gray matter loss, at least in the early stages of the disease. Dopaminergic disruptions may manifest alongside other markers of cognitive decline in PD which were not investigated in the present study (59).

### Strengths and limitations

Our study presents several notable strengths. By using a multimodal approach that integrates behavioral, cognitive, affective, neuroimaging and data-driven methods, we captured the complex presentation of non-motor symptoms in PD. In addition, by focusing on individuals with early-stage PD, our findings contribute to efforts aimed at identifying early markers of disease heterogeneity. Moreover, the use of a comprehensive cognitive battery allowed for the assessment of multiple cognitive domains, providing a broad and detailed picture of cognitive functioning in PD.

A few limitations to our study need to be addressed. First, the small number of PD participants who completed the PET/MR imaging may restrict our ability to draw firm conclusions about the neural correlates of symptoms profiles and underscores the need for further studies with larger imaging cohort. Even though the size of the full cohort was larger and of similar magnitude as in past phenotyping studies, the robustness of the data-driven LPA classification would probably have been improved by using larger sample size. The cross-sectional nature of the study limits the ability to infer causality or track symptom evolution over time. Accordingly, neurobiological interpretations in this study should be considered as associations rather than causal explanations. The observed relationship between dopaminergic integrity and NMS profiles generates hypotheses that require testing in prospective, longitudinal cohorts to establish temporal precedence and potential causal pathways. Finally, it is also important to note that we relied on self-reported measures for sleep and mood questionnaires, which may introduce reporting bias.

### Conclusion and implication of the current study

With this study, we revealed clinically distinct subgroups among individuals with early PD based on sleep disturbances, cognitive performance and neuropsychiatric symptoms. Notably, we observed a significantly impaired dopaminergic integrity in the left putamen in participants with greater NMS burden, highlighting a potential neurobiological signature underlying this specific phenotype. These findings underscore the importance of recognizing symptom heterogeneity early in the disease course, which may enhance our understanding of the pathophysiological mechanisms driving PD progression. Furthermore, early identification of such subgroups may inform more personalized clinical management and intervention strategies. Alongside the observed left putamen dopaminergic differences, functional imaging may provide a sensitive marker of subgroups differences in PD, especially in light of the behavioral distinctions observed, and will be the focus of future analyses in the iPARK project. Future research should aim to validate these subtypes longitudinally and explore their predictive value for disease progression.

## Data Availability

The datasets presented in this article are not readily available because of the ethical Review Act but is available from the corresponding author on reasonable request. Requests to access the datasets should be directed to AN, anna.stigsdotter.neely@ltu.se.
